# Comparison of the transcriptome in circulating leukocytes in early lactation between primiparous and multiparous cows provides evidence for age-related changes

**DOI:** 10.1186/s12864-021-07977-5

**Published:** 2021-09-25

**Authors:** Laura Buggiotti, Zhangrui Cheng, Mazdak Salavati, Claire D. Wathes, the Genotype plus Environment Consortium, Alan Fahey, Alessandra Crisà, Ali Fouladi, Alistair Wylie, Amelie Vanlierde, Anders Fogh, Andreia Santoro, Andrew Cromie, Anne-Sophie Van Laere, Armin Pearn, Arnold Evertson, Aurelie Laine, Beatriz Sanz Bernardo, Bianca Moioli, Bonny Vanranst, Catherine Bastin, Charlotte Gaillard, Chen Tan, Chris Elsik, Cinzia Marchitelli, Claire Wathes, Clement Grelet, Colin Byrne, Conrad Ferris, Daragh Matthews, Deborah Triant, Dirk Werling, Elizabeth Matthews, Else Meyer, Eric Froidmont, Federica Signorelli, Fiona Carter, Francesco Napolitano, Francis Kearney, Frank Becker, Frederic Colinet, Frederic Dehareng, Gavin Conant, Geert Opsomer, Geoff Pollott, Guiqiang Wang, Guohua Hua, Hannes Bogaert, Haruko Takeda, Hedi Hammami, Huanchun Chen, Jan Vandepitte, Janne Rothmann, Jehan Ettema, Jenne De Koster, Jennifer McClure, Jerry Taylor, Johanna Hoglund, Junlong Zhao, Klaus Ingvartsen, Kristof Hermans, Leila Vandevelde, Leslie Foldager, Liguo Yang, Linda Kosten, Luca Buttazzoni, Marilou Ramos Pamplona, Mark Crowe, Marlène Sciarretta, Martin Schulze, Martin Tang Sorensen, Matt Bell, Matt McClure, Matthew Lucy, Mazdak Salavati, Michel Bonneau, Michel Georges, Mieke Vaneetvelde, Miel Hostens, Mogens Krogh, Niamh McLoughlin, Nicolas Gengler, Pauline Rudd, Rodrigo Mota, Roisin O’Flaherty, Saied Naderi Darbagshahi, Sander Moerman, Sergio Palma Vera, Shujun Zhang, Sinead Hallinan, Soren Ostergaard, Susanne Dahl, Thomas Andersen, Tine Rousing, Torben Larsen, Victor H. Silva de Oliveira, Xing Chen, Zhangrui Cheng

**Affiliations:** 1 The Royal Veterinary College, Hawkshead Lane, Hatfield, Hertfordshire AL9 7TA UK; 2 The Roslin Institute, Royal (Dick) School of Veterinary Studies, Easter Bush Campus, Midlothian, UK

**Keywords:** Ageing, Leukocytes, Cow, Primiparous, Multiparous, Longevity

## Abstract

**Background:**

Previous studies have identified many immune pathways which are consistently altered in humans and model organisms as they age. Dairy cows are often culled at quite young ages due to an inability to cope adequately with metabolic and infectious diseases, resulting in reduced milk production and infertility. Improved longevity is therefore a desirable trait which would benefit both farmers and their cows. This study analysed the transcriptome derived from RNA-seq data of leukocytes obtained from Holstein cows in early lactation with respect to lactation number.

**Results:**

Samples were divided into three lactation groups for analysis: i) primiparous (PP, *n* = 53), ii) multiparous in lactations 2–3 (MP 2–3, *n* = 121), and iii) MP in lactations 4–7 (MP > 3, *n* = 55). Leukocyte expression was compared between PP vs MP > 3 cows with MP 2–3 as background using DESeq2 followed by weighted gene co-expression network analysis (WGCNA). Seven modules were significantly correlated (r ≥ 0.25) to the trait lactation number. Genes from the modules which were more highly expressed in either the PP or MP > 3 cows were pooled, and the gene lists subjected to David functional annotation cluster analysis. The top three clusters from modules more highly expressed in the PP cows all involved regulation of gene transcription, particularly zinc fingers. Another cluster included genes encoding enzymes in the mitochondrial beta-oxidation pathway. Top clusters up-regulated in MP > 3 cows included the terms *Glycolysis/Gluconeogenesis*, *C-type lectin*, and *Immunity.* Differentially expressed candidate genes for ageing previously identified in the human blood transcriptome up-regulated in PP cows were mainly associated with T-cell function (*CCR7*, *CD27*, *IL7R*, *CAMK4*, *CD28*), mitochondrial ribosomal proteins (*MRPS27*, *MRPS9*, *MRPS31*), and DNA replication and repair (*WRN*). Those up-regulated in MP > 3 cows encoded immune defence proteins (*LYZ*, *CTSZ*, *SREBF1*, *GRN*, *ANXA5*, *ADARB1*).

**Conclusions:**

Genes and pathways associated with lactation number in cows were identified for the first time to date, and we found that many were comparable to those known to be associated with ageing in humans and model organisms. We also detected changes in energy utilization and immune responses in leukocytes from older cows.

**Supplementary Information:**

The online version contains supplementary material available at 10.1186/s12864-021-07977-5.

## Background

Longevity is an economically important trait in dairy cows, which also has welfare implications. Cattle can potentially live for over 20 years, but this is rare in practice and the average lifespan in dairy cows is currently around 4.5 to 7 years [1–3]. To maximise economic potential, heifers should first calve at 24 months of age so becoming primiparous cows, starting their first lactation, and beginning to pay back their rearing costs through the production of saleable milk [4]. Ideally, they should continue to calve at annual intervals, with their milk production potential increasing progressively until the fourth or fifth lactation [5]. Cows which survive for longer achieve greater lifetime milk production associated with higher profitability [2, 3, 6] together with the benefit of reduced greenhouse gas emissions [7]. Many cows do not achieve optimum longevity due to premature involuntary culling, for which the main reasons are mastitis, infertility and lameness [8, 9]. On the other hand, voluntary culling may be used to remove cows with low milk yields or behavioural issues and to increase the rate of genetic gain [10].

There are major alterations in the metabolic profiles associated with the start of lactation [11]. These also change with age as primiparous cows are still growing, so their energetic demands for growth compete with those of milk production, whereas in older cows the higher milk yields are associated with greater mobilisation of body tissue, leading to higher circulating concentrations of nonesterified fatty acids (NEFA) and beta-hydroxybutyrate (BHB) [12, 13] Circulating concentrations of IGF1 are also significantly lower in multiparous (MP) than primiparous (PP) cows in early lactation [14]. IGF1 is a key metabolic hormone which provides a good indication of the energy balance status of the animal and is also linked to the immune responses following calving [15]. There are therefore important metabolic, endocrine and physiological changes that occur as cows mature, stop growing and increase their milk yields. Cows with greater milk production potential face an increased risk of glucose shortages in their immune cells, which contributes to immune dysfunction in the peripartum period [16]. Both metabolic and immune dysfunction therefore impact on the transcriptomic changes in leukocytes in early lactation.

Recent studies in human populations have highlighted age-associated changes in leucocyte functionality affecting innate and adaptive immune functions [17]. The causes and consequences of ageing on the human blood transcriptome have, however, proved difficult to dissect due to interactions with environmental influences, genetic factors and a large number of age-related diseases [18]. Studies on model organisms have highlighted that ageing is characterized by many alterations at molecular, cellular and tissue level [19]. Studies of the ageing transcriptome have been performed in species including *C. elegans* [20], flies [21], rodents [22] and humans [23]. This approach has identified various signatures found to occur repeatedly across different tissues and organisms. Candidate genes whose expression is consistently associated with cellular ageing have been classified into six categories: i) downregulation of genes encoding mitochondrial proteins, ii) downregulation of the protein synthesis machinery, iii) dysregulation of immune system genes, iv) a reduction in growth factor signalling, v) constitutive responses to stress and DNA damage, and vi) dysregulation of gene expression and mRNA processing [24].

Although all living creatures age at some point, our knowledge on the biology of ageing is still not sufficient. The physiological process of ageing in humans is associated with a progressive loss of function and increased vulnerability to disease, frailty, and disability [25]. Because the incidence of adult diseases increases with age, a better understanding of the biology of ageing could greatly improve our efforts to elucidate the physiopathology of such conditions [26].

In this study we have compared the leucocyte transcriptome between young (primiparous, PP) and older (multiparous, MP) cows in order to shed light on the genes and related genomic pathways involved in age-related symptoms arising during the different phases of a cow’s life. This has the potential to inform both breeding and management practices, so providing a significant gain to both agricultural production and animal welfare.

## Results

### Weighted gene co-expression network analysis (WGCNA) to determine the relationships of leucocyte gene transcription between cow parity

Whole blood transcriptomes of 229 cow samples from six dairy herds were obtained in early lactation at 14 ± 0.1 days in milk. The PP cows, as expected, produced less milk, but there was no difference in milk production between the MP2–3 and MP > 3 cows, with milk yields at the time of sample collection of PP 23.5 ± 0.88^a^ (*n* = 53); MP2–3 33.8 ± 0.68^b^ (*n* = 121) and MP > 3 33.0 ± 1.14^b^ (*n* = 55) kg/d respectively (mean ± SEM, b > a, *P* < 0.001; Supplementary Table 1). There was also a minor difference in milk production between two of the herds, with ITA cows producing slightly less and DEU cows slightly more milk (Supplementary Table 1). The herd effect was, however, accounted for in the WGCNA analysis by removing the batch effect (Supplementary Fig. 1).

**Fig. 1:**
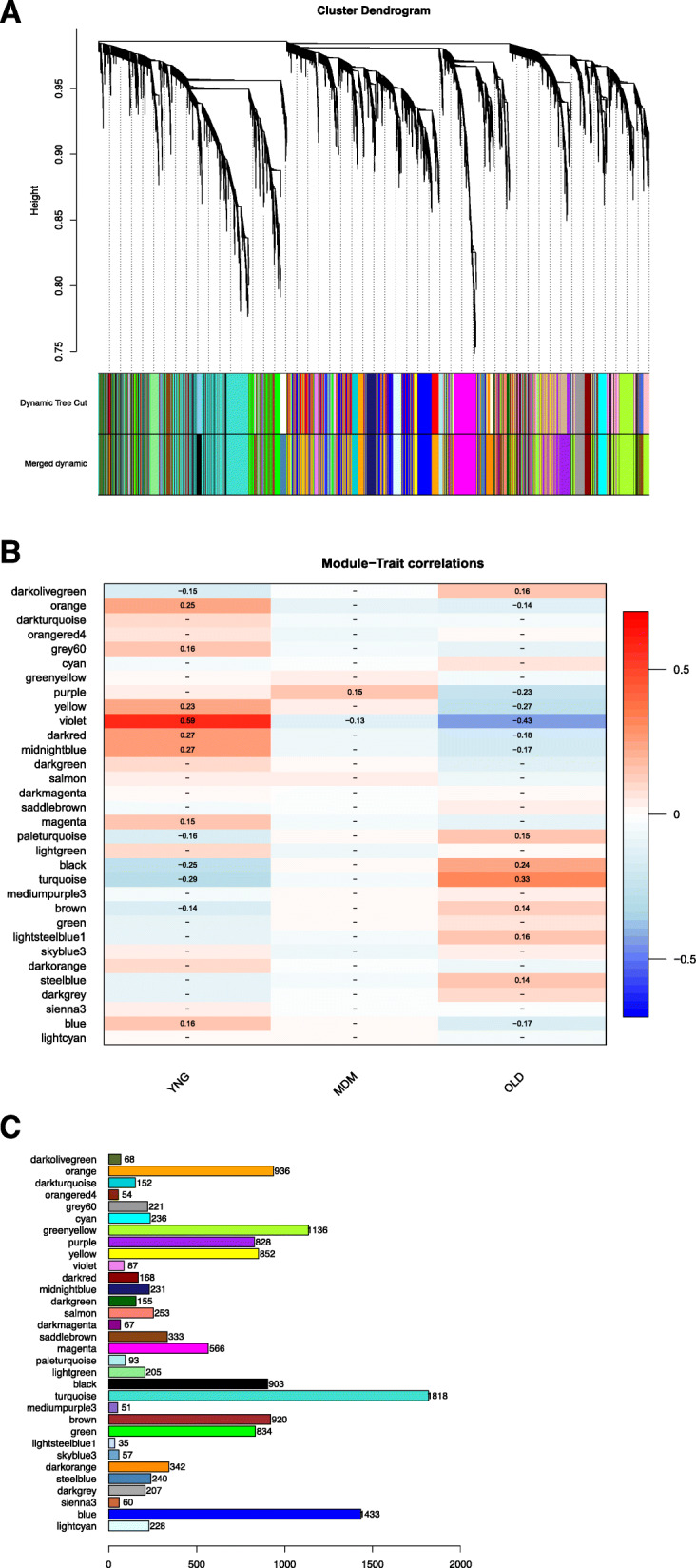
**A** Cluster dendrogram of 13,769 genes (individual black lines at top) clustered by their topological overlap dissimilarity scores. The multi-coloured panel next to “Dynamic Tree Cut” shows 42 identified modules using the Dynamic Tree Cut algorithm. The second multi-coloured panel shows 32 larger modules identified after highly correlated smaller modules were merged together (agreement of 0.8). **B** Number of genes per module identified using WGCNA. **C** Module-trait correlations according to the parity of the cow: i) primiparous (PP, *n* = 53), ii) multiparous in lactations 2–3 (MP 2–3, *n* = 121), and iii) MP in lactations 4–7 (MP > 3, *n* = 55). Red, positive correlation, blue, negative correlation. Numbers indicate the r value: significance was taken at *P* = ± 0.25

The leucocyte RNA samples had an average mapping rate against the reference genome of 96.2%, resulting in an average total number of reads of 34,439,525 (Supplementary Table 2). As we were most interested in understanding the effect of ageing, we contrasted the leukocyte expression between the two groups with extreme lactation numbers PP vs MP > 3, using those classified as MP2–3 as background. Firstly, we performed a differential expression analysis using DESeq2. Over 5000 genes out of the 17,216 genes initially mapped were found to be differentially expressed between lactation groups following Benjamini-Hochberg false discovery rate adjustment (padj < 0.1). This list was narrowed down to 2925 differentially expressed genes (DEG) when contrasting PP vs MP > 3 cows (Supplementary Fig. 2). We then applied WGCNA to identify gene modules from our dataset associated to the trait of lactation number. From an initial total of 21,207 genes, 13,769 (64.9%) genes passed the DESeq2 filtering steps. Samples from three cows were removed at this stage as outliers (two from MP > 3 and one from MP2–3 age group, respectively; Supplementary Fig. 3), leaving a total of 226 samples. This unsupervised technique identified 32 interconnected gene modules from the filtered gene list (Fig. 1A). The number of genes per module is shown in Fig. 1B. Of these, the violet and turquoise modules were significantly related to the trait lactation number in both the PP and MP > 3 groups (Fig. 1C). As expected, modules with a significant positive correlation in the younger cows were negatively correlated with lactation number in the older ones, and vice versa, while no modules showed a significant correlation with the medium lactation number group (MP2–3). Within these seven significant modules, 2274 genes were more highly expressed in the leukocytes of the PP cows and 2721 genes were more highly expressed in the leukocytes of the MP > 3 cows. The top 20 genes with the highest differential expression between PP and MP > 3 cows are listed in Table 1.

**Table 1:** Top 20 genes with the highest differential expression between PP and MP > 3 cows. Positive fold change (FC), more highly expressed in PP cows; negative FC (shaded), more highly expressed in MP > 3 cows

Gene Symbol	Gene name	logFC	adj.p.value
*NELL2*	neural EGFL like	−1.581	1.40E-63
*ZNF462*	zinc finger protein 462	1.931	1.79E-38
*PLCL1*	phospholipase C like 1 (inactive)	1.042	2.45E-37
*IGF2BP3*	insulin like growth factor 2 mRNA binding protein 3	2.268	6.36E-37
*CNTNAP1*	contactin associated protein 1	1.675	2.85E-35
*SOX13*	SRY-box transcription factor 13	1.925	8.17E-35
*BLK*	BLK proto-oncogene, Src family tyrosine kinase	0.881	1.23E-32
*MYBL1*	MYB proto-oncogene like 1	−0.771	1.93E-32
*CD163L1*	CD163 molecule like 1	1.463	6.55E-32
*WNT5A*	Wnt family member 5A	2.673	2.56E-31
*SCRN1*	secernin 1	1.129	5.03E-31
*LOC515828*	uncharacterised	1.207	6.12E-30
*CSPG4*	chondroitin sulfate proteoglycan 4	2.831	8.30E-30
*DBN1*	drebrin 1	0.942	1.95E-29
*PTPRK*	protein tyrosine phosphatase receptor type K	−3.301	2.00E-29
*LAMA4*	laminin subunit alpha 4	2.998	4.15E-29
*TCAF1*	TRPM8 channel associated factor 1	1.050	1.17E-28
*LOC751811*	WC1 isolate CH211	1.389	2.85E-28
*SOX4*	SRY-box transcription factor 4	0.820	3.69E-28
*LOC104969122*	uncharacterised	−1.396	9.97E-28

### Significant modules in leukocytes of both PP and MP > 3 cows

**Fig. 2:**
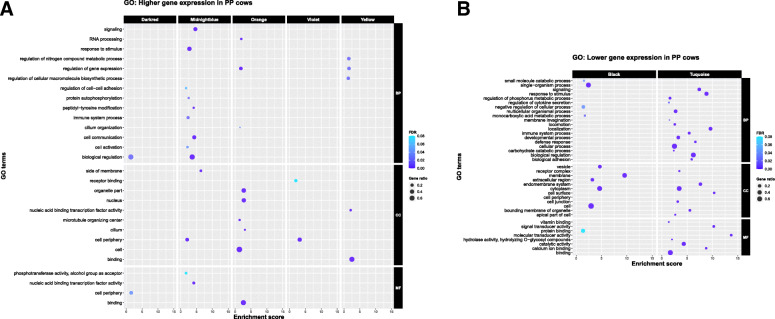
GO enrichment analysis of significantly correlated modules (FDR < =0.1). **A** Positively correlated in PP cows: violet, darkred, midnightblue, orange, yellow; **B** negatively correlated in PP cows: black, turquoise

The violet module containing 87 genes had the highest correlations with lactation number (+ 0.59 in PP and − 0.43 in MP > 3 cows, Fig. 1C). Within this module the *LAMA4* gene was the most highly correlated gene (Table 1, gene significance = 9.05 × 10^− 22^), with lower expression in the MP > 3 cows, representing its module membership. This is a laminin gene, part of the family of extracellular matrix glycoproteins which form the major non-collagenous constituent of basement membranes. GO enrichment analysis of the genes in this module revealed the terms *receptor binding* and *cell periphery* (Fig. 2A).

The turquoise module represents the second module which had significant correlations in opposite directions with PP and MP > 3 cows, of − 0.29 and 0.33, respectively. This module contained 1818 genes of which *NELL2* was the most highly correlated with lactation number (Table 1). This was also overall the most differentially expressed gene in leucocytes with higher expression in MP > 3 cows. It was a large module containing 1818 genes with a wide variety of functions (Fig. 2B, Supplementary Table 3). The top six most significant GO terms (all < 1.36 × 10^− 25^ and containing between 389 and 959 genes) were *extracellular region, membrane, single-organism process, vesicle* and *cell periphery*.

### Modules up-regulated in leukocytes of PP cows

The modules midnightblue, darkred, and orange were all significantly positive in PP cows with correlations spanning from 0.27 to 0.25, implying lower expression in leukocytes as lactation number increased (Fig. 2A, Supplementary Table 3). The most significant GO terms in the midnightblue module were all biological processes: *protein autophosphorylation, immune system process, cell activation* and *regulation of cell-cell adhesion*. The darkred module had enrichment of biological regulation and the molecular function term *cell periphery*, while the orange module was mainly enriched with genes involved in *cilium* and *cilium organisation; RNA processing and regulation of gene expression*, and *microtubule organizing center*.

### Black module: down-regulated in leukocytes of PP cows

The black module was down-regulated in leukocytes of PP (correlation of − 0.25) and almost significantly up-regulated in MP > 3 (correlation of 0.24). Genes were therefore more highly expressed in the older cows with higher lactation numbers. GO enrichment analysis of genes in this module (Fig. 2B, Supplementary Table 3) showed that the most significant terms all related to cellular components (*membrane, vesicle, cytoplasm, extracellular region* and *cell*). Within the biological process category *single-organism process, monocarboxylic acid metabolic process, small molecule catabolic process* and *negative regulation of cellular process* were significant.

### Yellow module: down-regulated in leukocytes of MP > 3 cows

Lastly, the yellow module was significantly down-regulated in leukocytes as lactation number increased (MP > 3; correlation − 0.27). In this module *IGF2BP3* (insulin like growth factor 2 mRNA binding protein 3) was the most highly correlated gene (Table 1). Within this module the two cellular component terms *binding* and *nucleic acid binding transcription factor activity* were most highly represented, together with three biological processes (*regulation of gene expression, regulation of nitrogen compound metabolic process* and *regulation of cellular macromolecule biosynthetic process*) (Fig. 2A, Supplementary Table 3).

### Functional annotation cluster analysis

The next stage of the analysis involved pooling all the genes from the modules which were more highly expressed in the PP cows (yellow, orange, midnightblue, darkred, violet) and those which were more highly expressed in the MP > 3 cows (black and turquoise). These two gene lists were then subjected to David functional annotation cluster analysis [27]. All the results are provided in Supplementary Table 4. The top six most significant clusters in each category are summarised in Table 2, with the genes in each of these clusters listed in Supplementary Tables 5A and B.

**Table 2:** Summary of the six most significant clusters obtained from DAVID functional annotation cluster analysis of the DEG in the modules which were significantly positively or negatively correlated to lactation number in primiparous (PP) cows, indicating either lower or higher gene expression in older multiparous MP > 3 cows

Positive Modules	Negative Modules
**Cluster 1: Enrichment Score: 16.18**	**Cluster 1:Enrichment Score: 29.73**
• Krueppel-associated box • Zinc finger C2H2-type/integrase DNA-binding domain • Zinc finger, C2H2 • Nucleic acid binding • Zinc finger, C2H2-like • KRAB • ZnF_C2H2 • Regulation of transcription, DNA-templated • Metal ion binding	• Membrane • Transmembrane helix • Transmembrane • integral component of membrane
**Cluster 2: Enrichment Score: 7.01**	**Cluster 2: Enrichment Score: 19.87**
• Zinc-finger • Metal ion binding • Zinc • Metal-binding	• Signal • Disulfide bond • Glycoprotein • Signal peptide • Glycosylation site: N-linked (GlcNAc...) • Disulfide bond
**Cluster 3: Enrichment Score: 6.83**	**Cluster 3: Enrichment Score: 7.32**
• Transcription regulator SCAN • Retrovirus capsid, C-terminal • SCAN	• Transmembrane region • Topological domain: Cytoplasmic • Topological domain: Extracellular
**Cluster 4: Enrichment Score: 5.05**	**Cluster 4: Enrichment Score: 3.94**
• Nucleotide binding • Nucleotide-binding, alpha-beta plait • RNA recognition motif domain • RRM	• Glycolysis / Gluconeogenesis • Glycolysis • Glycolytic process • Carbon metabolism • Biosynthesis of amino acids
**Cluster 5: Enrichment Score: 2.02**	**Cluster 5: Enrichment Score: 3.70**
• BTB/POZ fold • BTB/POZ-like • BTB	• C-type lectin fold • C-type lectin-like • C-type lectin • CLECT • C-type lectin, conserved site
**Cluster 6: Enrichment Score: 1.88**	**Cluster 6: Enrichment Score: 3.28**
• Immunoglobulin-like domain • Immunoglobulin-like fold • Immunoglobulin subtype 2 • Immunoglobulin I-set • Immunoglobulin subtype • IGc2 • IG	• Immunity • Innate immunity • Innate immune response

Clusters 1, 2 and 3 from the modules which were positively correlated in the PP cows were all involved in the regulation of gene transcription. Cluster 1 consisted almost entirely of 56 genes encoding zinc finger proteins. The most significant term in Cluster 1 was *Krueppel-associated box*. All but five of the Cluster 1 genes were included in the much larger Cluster 2 with 142 genes, for which the most significant terms were *Zinc-finger* and *metal ion binding*. Cluster 2 also contained all 18 genes from Cluster 3, for which the three significant terms were *Transcription regulator SCAN*, *Retrovirus capsid, C-terminal* and *SCAN*. Cluster 4 contained 61 genes involved in *nucleotide binding*. Cluster 5 contained 31 genes for which the most significant term was *BTB/POZ fold*. Finally, Cluster 6 contained 50 genes with the most significant term being *Immunoglobulin-like domain*. Overall, this analysis shows that the most significant functions which were more highly expressed in the first lactation cows all related to the regulation of gene transcription.

**Fig. 3:**
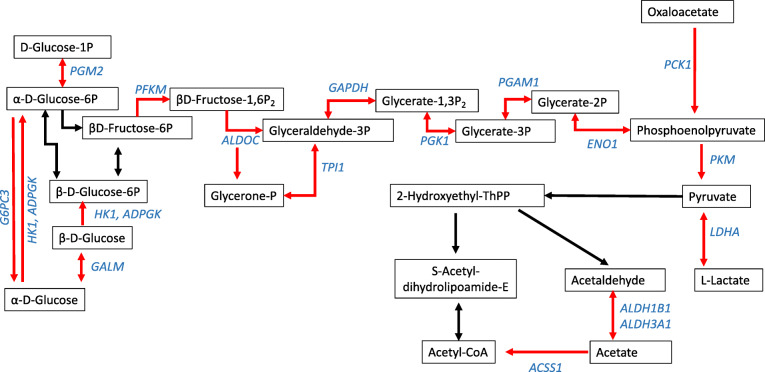
David functional annotation Cluster 4 contained many genes (shown in blue) in the Glycolysis/Gluconeogenesis KEGG pathway which were more highly expressed in MP > 3 vs PP cows (figure drawn by the authors). This would potentially maximise the use of glucose as a precursor of acetyl CoA for use in the TCA cycle

Turning to the genes which were more highly expressed in the MP > 3 cows, Clusters 1, 2 and 3 were all large clusters containing 471, 435, and 254 genes, respectively, with quite general functions relating to the cell surface, transport across it and signalling. In Cluster 1 the top three terms were *Membrane*, *Transmembrane helix* and *Transmembrane*, in Cluster 2 they were *Signal*, *Disulfide bond* and *Glycoprotein* and in Cluster 3 *transmembrane region*, *topological domain: Cytoplasmic* and *topological domain: Extracellular*. The top term in Cluster 4 was *Glycolysis/Gluconeogenesis* and this contained 27 genes, nearly all of which encoded enzymes involved in the glycolytic pathway (Fig. 3).

**Fig. 4:**
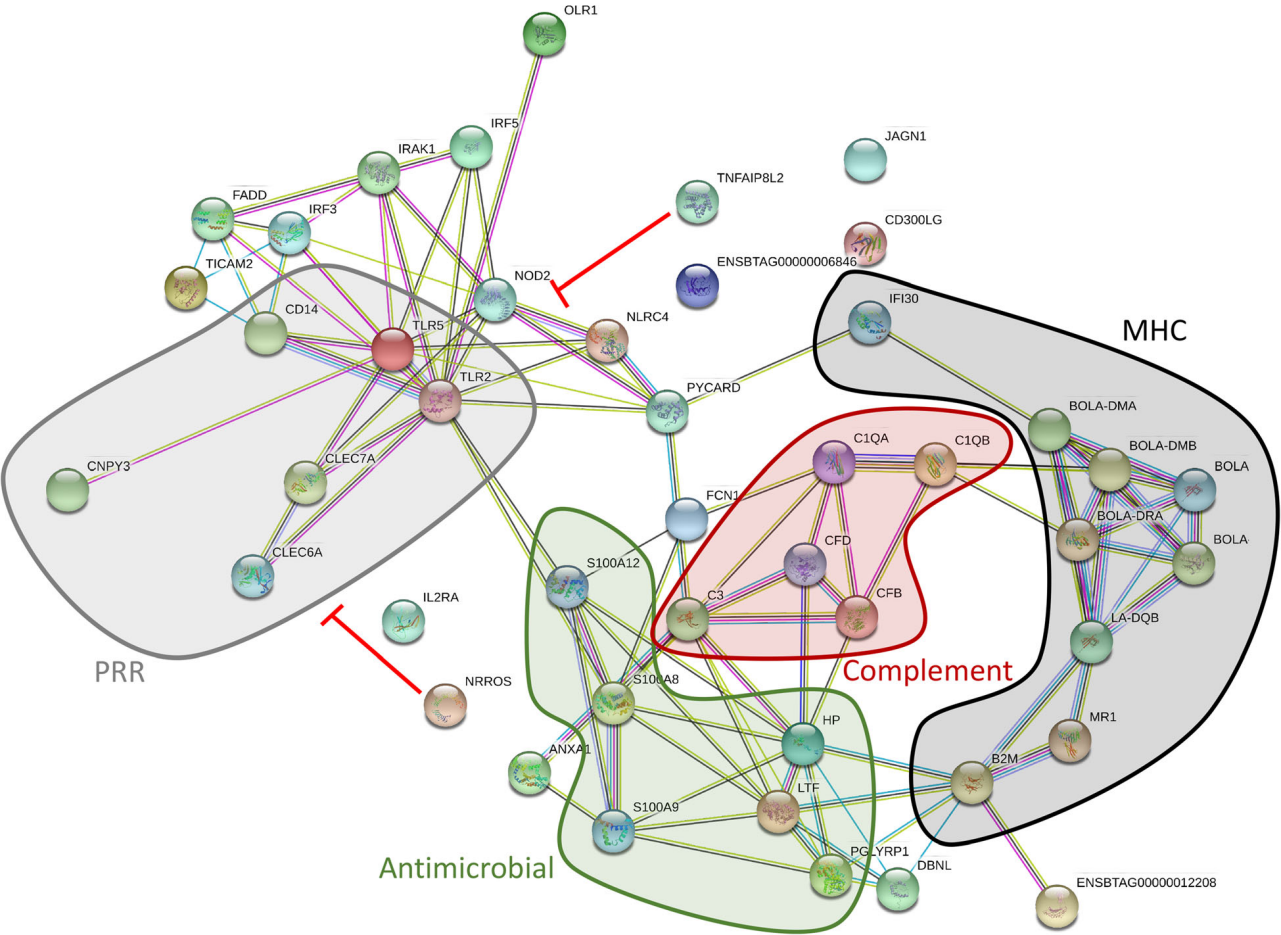
David functional annotation Cluster 6 contained genes involved in immunity which were more highly expressed in MP > 3 vs PP cows. This included genes encoding proteins for pattern recognition receptors (PRR), the bovine MHC complex, the complement system and several with antimicrobial activity. NRROS and TNFAIP8L2 can both act as negative regulators of TLR signalling mediated via inhibition of NF-kappa-B activation

The top term in Cluster 5 was *C-type lectin fold*, and 11 of the 25 genes in this cluster encoded C-lectins. Finally, Cluster 6 contained 45 genes involved in *Immunity* and *Innate immunity*. These are illustrated in Fig. 4, which shows that they mainly encoded proteins for pattern recognition receptors (PRR), the bovine MHC complex, the complement system and several with antimicrobial activity. Another noteworthy cluster which was negatively related to lactation number was Cluster 16 (Supplementary Table 4). The top pathway in this was *Fatty acid degradation* (*P* < 0.0013). This cluster contained a number of genes encoding enzymes in the mitochondrial beta-oxidation pathway, which converts fatty acids into acetyl CoA to enter the TCA cycle (*ACSL1, ACSL4, ACSL5, ACSL6, CPT1, CPT2, ACOX1, ACADVL, ECHS1, ACAD5, ACAA1, ACAT2, ACADS, ACADVL*). This implies greater use of this pathway to supply energy in the leukocytes of the PP cows.

### Comparison with other species

Finally, we investigated the overlap between genes significantly associated with lactation number in leucocytes in our study and 170 candidate genes identified from previous studies of ageing in humans and model organisms (mainly mice and *C. elegans* [18]). Almost one quarter of these candidate genes (38/170, 22.3%) were present in our list of DEG, of which 14 and 24 respectively were from positive and negative significantly correlated modules, indicating reduced or increased expression in older cows. Of these 16 of the 38 DEG were previously identified as being age-related in the human blood transcriptome ([18]; Table 3).

**Table 3:** List of genes overlapping candidate age-related genes found in humans and with differential expression between PP (highly expressed) and MP > 3 (highly expressed) cows

	Gene name	Module	Description	Reference
**PP**	CCR7	darkred	Control the migration of memory T cells to inflamed tissues, as well as stimulating dendritic cell maturation	[28]
	CD27	midnightblue	Required for generation and long-term maintenance of T cell immunity	[28]
	FOXO1	midnightblue	Transcription factor which may play a role in myogenic growth and differentiation	[29]
	IL7R	midnightblue	Plays a critical role during lymphocyte development (age and longevity expression association)	[30]
	CAMK4	midnightblue	Implicated in transcriptional regulation in lymphocytes, neurons and male germ cells (longevity genetics candidate)	[31]
	CD28	midnightblue	Essential for T-cell proliferation and survival, cytokine production, and T-helper type-2 development	[28]
	WRN	yellow	RecQ helicase important in the maintenance of genome stability. Mutations cause Werner syndrome (premature ageing)	[32]
	MRPS27	yellow	Mitochondrial ribosomal protein assisting protein synthesis within the mitochondrion (mitochondrial ageing)	[33]
	MRPS9	yellow	Mitochondrial ribosomal protein assisting protein synthesis within the mitochondrion (mitochondrial ageing)	[33]
	MRPS31	orange	Mitochondrial ribosomal protein assisting protein synthesis within the mitochondrion (mitochondrial ageing)	[33]
**MP > 3**	SREBF1	black	Transcription factor involved in sterol biosynthesis	[29]
	ANXA5	turquoise	Potential role in cellular signal transduction, inflammation, growth and differentiation	[34]
	LYZ	turquoise	Antimicrobial peptide	[34]
	CTSZ	turquoise	Lysosomal cysteine protease with role in immune defense	[34]
	ADARB1	turquoise	Enzyme in the A (adenosine) to I (inosine) RNA editing pathway, involved in a general maintenance of cellular health (longevity genetics candidate)	[35]
	GRN	turquoise	Granulin precursor protein which regulates cell growth. Also, co-ordinates CpG trafficking to TLR9, promoting activation of signalling cascade	[34]

Those up-regulated in PP cows were mainly associated with T-cell development and function (*CCR7*, *CD27*, *IL7R*, *CAMK4*, *CD28*) and protein synthesis within the mitochondria (*MRPS27*, *MRPS9*, *MRPS31*), and also included WRN, a gene associated with premature ageing in humans [36]. Those up-regulated in MP > 3 cows included genes encoding proteins involved in immune defence (*LYZ*, *CTSZ*, *SREBF1*, *GRN*, *ANXA5* and *ADARB1*).

## Discussion

Livestock species are raised primarily for their economic benefit to humans. Most dairy cows are culled before they reach the end of their potential lifespan due to poor milk production or fertility and/or an increased prevalence of diseases such as those causing mastitis or lameness [3, 8, 9]. Various cow longevity indexes have been defined, some of which also take account of lifetime milk production, which is in turn affected by both the milk yield capacity and the number of lactations achieved [3]. The main focus of previous studies into longevity in cows has been to increase the average survival time in the milking herd, so improving the profitability of the dairy industry. For example, one genome wide association study (GWAS) which investigated longevity found genes such as *NPFFR2*, previously identified as a candidate gene for mastitis resistance and two zinc finger proteins (*ZNF613, ZNF717*), which have been associated with calving difficulties [37]. Information about the age-related morbidities and causes of death that afflict cattle due to natural ageing is, however, limited. In contrast, there is a growing body of previous work into the underlying causes of cellular ageing which has been based on studies of human populations and model organisms.

This study is the first, to our knowledge, to assess changes in the global gene expression in leukocytes of dairy cows associated with increasing lactation number. For this we compared first lactation PP cows versus older multiparous MP > 3 cows using WCGNA analysis in order to identify potential genes and related pathways involved in the ageing process. The blood samples were all collected in early lactation, a time when lactating cows are placed under metabolic stress due to the nutrient requirements of milk production [11, 38] but none are pregnant. These aspects improved comparability between samples but meant that we did not include any data from younger growing heifers of < 2 years. The general aim of most dairy farmers is to calve animals for the first time at 2 years of age and then to achieve an annual calving interval, although in practice these targets are rarely met [39]. Although we did not record the exact ages of the cows in this study, the expectation would be for the PP cows to have been between 2 and 2.5 years old, the MP2–3 between 3.5–6 years old and the MP > 3 (which were in lactations 4–7) would have been between 6 and 10 years old. In most countries worldwide, dairy cow longevity has declined over the past 50 years and has been negatively related to the rise in milk yields achieved over the same period [3]. Despite the many differences in physiology, lifestyle and lifespan between species, many of the genes and pathways identified in our study as being associated with increased lactation number in dairy cows were nevertheless the same as those highlighted in previous studies of ageing in other species [18–20, 23].

All the samples analysed were whole blood leukocytes. In dairy cows the average leucocyte population is between 5 and 12 cells per ml of blood, predominantly consisting of lymphocytes and neutrophils [40]. The blood does, however, also contain natural killer cells, platelets, peripheral blood mononuclear cells (PBMC), eosinophils, and basophils. These different cell types all vary in both their basal gene expression and their transcriptional amplification responses to particular stimuli [41]. Furthermore, many aspects of the immune system alter during ageing, eventually leading to immunosenescence [24]. During this process different immune cell subsets are affected in different ways [17]. There is also an increase in baseline systemic inflammation with age, termed “inflammaging” [42, 43]. This may arise as a result of multiple mechanisms, including the accumulation of misfolded proteins, impaired clearance of senescent cells and obesity. Most individual leukocytes have a short lifespan in the circulation, measured in days or weeks, before they are destroyed by the lymphatic system, although there is a small pool of long-lived T and B lymphocytes which can survive for years, providing immunological memory. In humans, the relative abundance of naive T-cells decreases with chronological age while the population of memory T-cells increases [18, 44, 45]. Another possible source of variation in our analysis was the potential differences between herds, but this was accounted for by removing the batch effect in the WGCNA. Two of the six herds also fed three different lactational diets. Although these did influence cow metabolism this was not yet apparent at Day 14 when the samples used in the present study were collected. A comparison of the transcriptome in PBMC collected from the same UK herd failed to detect any DEG attributable to diet at this time [46].

The leukocyte transcriptome thus provides a reflection of the basic functions required for cell survival together with the various responses of the different cell types to the changing environment within the body, which alters with key factors such as disease exposure and nutrition. Cells may proliferate, undergo apoptosis or migrate to or from tissues into blood in response to different signals [47]. The changes reported here which were associated with lactation number will, to some extent, reflect altered abundance of different cell types as well as their changing expression patterns. Despite this caveat, many previous studies have now reported that transcriptional signatures of whole blood can reliably differentiate individuals with a variety of infections, for example Johne’s disease (*Mycobacterium avium subsp. Paratuberculosis*) in cattle [48]. Our analysis also benefitted from the use of WGCNA, which identifies networks of co-expressed genes whose expression is highly correlated. The use of whole blood also avoided the pitfall of potential artefacts which can be induced during cell separation procedures and provided greater consistency in a multi-site study.

Previous work based on studies of human populations and model organisms has identified a number of transcriptional signatures for cellular ageing which occur repeatedly across different tissues and organisms and which segregate into six main groups [24, 49]. In brief these are: i) downregulation of genes encoding mitochondrial proteins; ii) downregulation of the protein synthesis machinery (including ribosome biogenesis); iii) dysregulation of immune system genes, immune senescence; iv) reduction in growth factor signalling; v) constitutive responses to stress and accumulated DNA damage, and vi) dysregulation of processes regulating gene expression and mRNA processing (transcription and translation). We have obtained evidence that all of these were associated with lactation number within our population of cows.

### Mitochondria and oxidative stress

Mitochondria regulate a multitude of different metabolic and signalling pathways and also play an important role in programmed cell death [50]. Oxidative metabolism causes endogenous production of free radical molecules and oxidative damage accumulates in multiple tissues and species with age [49]. For example, accumulated mutations in somatic mitochondrial DNA (mtDNA) and respiratory chain dysfunction were associated with ageing in mice [51]. In our study, genes encoding proteins involved in fatty acid beta-oxidation were more highly expressed in leukocytes in PP cows. There were also changes in expression of genes encoding mitochondrial ribosomal proteins, with higher expression of *MRPS9, MRPS27* and *MRPS31* in leucocytes of PP cows whereas *MRPL17* and *MRPL38* showed enriched expression in MP > 3 cows**.**

### Protein synthesis

Protein homeostasis is essential to maintain protein structure and function, but the control of this process declines during ageing [49]. In our study, GO enrichment analysis of all the genes in the violet module, which were more highly expressed in the first lactation cows, revealed their involvement in *protein autophosphorylation* and *catalytic activity* (enzyme activity). Autophosphorylation is a type of post-translational modification of proteins. In eukaryotes, this process occurs by the addition of a phosphate group to serine, threonine or tyrosine residues within protein kinases, normally to regulate the catalytic activity. Genes involved in *regulation of nitrogen compound metabolic process* were identified in the yellow module as being down-regulated in the older MP > 3 cows. Maintenance of the proteome is essential to enable cells to respond appropriately to their environment. This requires correct synthesis and assembly of proteins and is controlled by molecular chaperones and clearance mechanisms which help to prevent protein misfolding and the associated accumulation of toxic aggregates. The efficiency of this process declines with age and has previously been associated with both metabolic and immunological diseases [49, 52].

### Immune system

As we were studying a leukocyte population, changes in gene expression relating to immune function were expected. As animals age they are exposed to an increasing variety of disease causing microorganisms, while a progressive loss of function of the immune system increases their vulnerability to infection [25]. Notable age-related changes within the immune cell population include reduced cytokine signalling, diminished production of nitric oxide and peroxide, decreased phagocytic ability and reduced ability of dendritic cells to migrate and process antigens [17]. Our results are in accord with the study by Peters et al. [18], who investigated leucocytes from ageing human populations, in finding pathways which were either up- or down-regulated with increasing age. The GO term *immune system process* was enriched in both PP and MP > 3 cows, with genes involved in adaptive immunity up-regulated in the PP cows (*FYN*, *ITK*, *LCK*, etc) and genes related to innate immunity up-regulated in MP > 3 cows (*CTSS*, *CTSH*, *IRAK2*, *TLR2*, etc). The black module was down-regulated in the PP cows and contained genes involved in the *regulation of cytokine secretion* (*IL10*, *CD14*, *FGR*, *IL17RC).* Expression of genes in the turquoise module increased in older cows, containing genes involved in *neutrophil degranulation* and *innate immune system*. The darkred and midnightblue modules were both more highly expressed in the PP cows and contained the terms *disease* and *immune system*.

There was also a significant overlap between the DEG with immune functions identified in our population of cows and candidate age-related genes in the human transcriptome [18]. Those up-regulated in PP cows were mainly associated with T-cell development and function (*CCR7, CD27, IL7R, CAMK4, CD28)* while those up-regulated in MP > 3 cows included genes encoding proteins involved in immune defence. Of these *LYZ* encodes lysozyme, an antimicrobial peptide, CTSZ encodes cathepsin Z a lysosomal cysteine protease with multiple roles in host immune defense mechanisms, *SREBF1* encodes a transcription factor involved in TLR4 signalling, while *GRN* encodes granulin, involved in TLR9 signalling, *ANXA5* is involved in T-cell activation and *ADARB1* encodes a deaminase enzyme with A-to-I RNA editing activity, which is important for the maintenance of cellular health but may also play a role in response to viral infection. The results from our study therefore suggest that the higher lactation number cows are more actively engaged in combatting disease pathogens through activation of the innate immune system and also support a higher level of inflammation with ageing. Parturition is itself an inflammatory process and, in addition to higher rates of infectious diseases including mastitis and metritis, older cows are also more vulnerable to metabolic disorders including milk fever, ketosis and displaced abomasum after calving. The prevalence of all of these metabolic diseases increased significantly in cows which were ≥ 5 lactations [53]. Our previous study showed that metabolic disorders led to prolonged uterine inflammation by up-regulating the genes and pathways related to immune and inflammatory processes [15].

### Growth factor signalling

The relationship between ageing and metabolic regulation is bidirectional, as ageing impairs the activity of key metabolic signalling pathways and the ensuing metabolic dysregulation results in accelerated ageing [19]. Cell signalling pathways that sense the availability of nutrients and the energy status of the cells communicate with hormonal and growth factor signalling pathways to co-ordinately regulate whole body metabolic homeostasis. Ageing results in a gradual deterioration of various cellular functions including metabolic regulation [54]. The turquoise module was the second highest correlated module with lactation number, containing genes more highly expressed in MP > 3 cows. The *insulin-like growth factor binding protein* pathway was indeed enriched with genes like *INSR*, *IGF1R*, *IGF1*, *LDLR, HTRA1, IGFBP7* etc. (Cluster 8; Supplementary Table 4). The latter pathway is interlinked with metabolic pathways to ensure coordinate regulation and fine-tuning of cellular metabolic responses in line with cellular energy status, nutrient availability and hormonal/growth factor signalling input [54]. We have shown previously that circulating IGF1 concentrations are significantly lower in PP compared with MP cows, falling to a lower nadir in the first week after calving [14].

### Stress and DNA damage

Accumulation of genetic damage represents one of the major contributions to ageing of cells and organisms. Cellular DNA is constantly exposed to exogenous and endogenous DNA-damaging agents like reactive oxygen species, nitric oxide metabolites, and alkylating agents [55] leading to accumulation of mutations in the genome aggravated by loss of capacity in the DNA repair systems [56]. DNA damage is tightly linked to various ageing stresses, such as oxidative stress, telomere shortening, inflammation, irradiation, exposure to chemicals, and mitotic stress [57]. This is supported by a recent study which took repeated measurement of the relative leukocyte telomere length in a dairy herd. Higher rates of telomere attrition in individual cows was predictive of a shorter productive lifespan, suggesting a link between telomere loss and health [58].

In our dataset, *NELL2* was the gene most highly correlated with lactation number in the turquoise module and was also overall the most differentially expressed gene between the PP and MP > 3 cows, with greater leucocyte expression in the older animals. The encoded protein Neural EGFL Like 2 is highly conserved in mammals and is a glycoprotein containing several von Willebrand factor C and epidermal growth factor (EGF)-like domains. This has a variety of possible roles but amongst these a cell survival-promoting effect mediated by an intracellular mitogen-activated protein kinase (MAPK) pathway has been relatively well studied in neural tissues [59, 60]. NELL2 is also important in protecting cells from death caused by endoplasmic reticulum (ER) stress resulting in the accumulation of unfolded proteins which trigger the unfolded protein response. Within this context, overexpression of *NELL2* decreased expression of ER stress-induced C/EBP homologous protein (CHOP) and the pro-apoptotic caspases 3 and 7 while increasing expression of ER chaperones and anti-apoptotic Bcl-xL [61].

Another relevant gene to consider is *MTOR,* which encodes Mechanistic Target of Rapomycin, a protein belonging to a family of phosphatidylinositol kinase-related kinases which mediate cellular responses to stresses such as DNA damage and nutrient deprivation. This gene is a central regulator of metabolic homeostasis and is associated with lifespan in many species [62, 63]. MTOR is a component of two distinct complexes of which mTORC1 controls protein synthesis, cell growth and proliferation. Genes which are part of the MTOR pathway include the transcription factors *FOXO1* and *FOXP1*. A GWAS for longevity, based on a population of Holstein cows, previously identified a region on Bta16 containing MTOR [64]. In our study *FOXP1* expression was positively related to increasing lactation number, whereas *FOXO1* expression was negatively related. Also, of potential relevance here is the opposing roles of these two FOX genes in the regulation of glucose homeostasis, through competition in binding to the insulin response element in gene promoters. Up-regulation of *FOXP1* in mice inhibited the hepatic expression of key gluconeogenic genes, including *PGC-1α*, *PEPCK* and *G6PC* [65]. *LAMTOR1, − 2* and *− 3* all also featured in the list of DEG which were negatively related to lactation number in our study. These genes encode late endosomal/lysosomal adaptor, MAPK and MTOR activator-1, − 2 and − 3 respectively, all subunits of the Ragulator complex. This functions as a lysosome anchor, which recruits Rag GTPase and its associated mTORC1 complex to the lysosomal surface prior to MTOR activation [66].

### Regulation of gene expression

The control of gene expression becomes more dysregulated with cellular ageing. A large number of genes and pathways identified in this study are involved in regulation of gene expression. The violet module was the most highly correlated with lactation number and contained a group of genes involved in transcriptional regulation including *ZNF462*, *SOX13*, and *SOX4,* all of which featured individually in the top 20 genes whose expression was most highly correlated with age (Table 1). Genes in the orange module were more highly expressed in PP cows and this module was enriched with genes involved in *gene expression* (*CHTOP*, *CPSF6*, *THOC1*, *UPF3B*, etc.) and *metabolism of RNA* (*AMDHD1*, *SRSF2*, *SRSF4*, *SRSF5*, etc.). In the yellow module *IGF2BP3* was down-regulated in the older cows and was the most highly correlated gene. IGF2BP family members were initially identified as post-transcriptional regulators of IGF2. They are RNA-binding proteins which direct nuclear RNA export and translation/degradation rates, so playing a major role as regulators of the RNA life cycle. *IGF2BP3* has recently risen to prominence as a potential oncogene [67]. Other genes in this module were also grouped under *regulation of gene expression* (*TGFB3*, *NEUROG2*, *ZNF554*, etc.). The yellow module also contained the gene *WRN,* which exhibited lower transcript abundance in MP > 3 cows. A mutation in this gene in humans causes Werner Syndrome, an autosomal recessive disorder characterized by the premature development of ageing features. The encoded protein is a member of the RecQ family of proteins and is involved in DNA replication and repair, and telomere maintenance, so playing a crucial role in genome stability [36]. Expression of *WRN* was similarly reduced in leucocytes of older humans [18]. *ADARB1* was another candidate gene from previous studies which was up-regulated in the older MP > 3 cows. It encodes a deaminase enzyme with A-to-I RNA editing activity, was previously identified in a study of men aged 90–119 years, and is also associated with longevity in *C. elegans* [35].

### Metabolism

One key difference between PP and MP > 3 is that milk production potential in dairy cows increases with age [5]. The liver coordinates the extensive metabolic changes required for milk production and these are reflected in circulating metabolite concentrations [11, 38]. Milk synthesis has a high requirement for glucose. In ruminants this demand is met almost exclusively through hepatic gluconeogenesis and cows are at risk of glucose insufficiency during early lactation, the time period we investigated [16]. NEFA are released from lipid stores as an alternative energy source and are either used by the udder to provide milk triglycerides, fully oxidized in the liver to provide energy, or partially oxidised resulting in the production of ketone bodies, in particular BHB. Circulating BHB concentrations are thus an index of fatty acid oxidation and concentrations are significantly higher in older cows [12]. BHB, NEFA and glucose concentrations can all influence leukocytes directly. Immune cells require an adequate supply of nutrients including glucose to mount an effective immune defense [68]. Neutrophils from cows with more elevated NEFA and BHB concentrations after calving had reduced expression of genes important for granulocyte recruitment, IFN signaling and apoptosis [69]. This suggested that neutrophil survival time was longer in the circulation when exposed to pro-inflammatory conditions. Another study of the circulating leucocyte transcriptome in early lactation found that expression of genes in KEGG pathways relating to DNA replication, cell cycle, homologous recombination, base excision repair, and valine, leucine, and isoleucine biosynthesis were all inhibited as plasma BHB increased, whereas genes involved with vitamin metabolism, the endocrine system, signalling molecules and the immune system were activated [70].

In this study genes in both black and turquoise modules were negatively significantly correlated to cow parity and David functional annotation cluster analysis found enrichment of the terms *fatty acid metabolism* and *glycolysis*. Interestingly, we found that the majority of 27 genes in Cluster 4, with higher expression in MP > 3 cows, were involved in the *Glycolysis/Gluconeogenesis* pathway (Fig. 3). Although the underlying metabolic background is very different, up-regulation of a small cluster of genes relating to “Fatty acid metabolism, peroxisome activity” was also associated with ageing in the human blood transcriptome [18].

### Other key genes associated with lactation number

Within the violet module *LAMA4* was the most highly correlated gene overall, with greater expression in the PP cows. This encodes a subunit of laminin, part of the family of extracellular matrix glycoproteins which form the major non-collagenous constituent of basement membranes. Laminin is thought to mediate the attachment, migration and organization of cells into tissues by interacting with other extracellular matrix components, suggesting that these activities may be reduced in older cows. There is a second MTOR complex mTORC2, which acts as a regulator of the actin cytoskeleton. This is a network of actin and actin binding proteins which are important for a range of essential cellular processes including organelle transport, cell migration, phagocytosis, and cell cycle progression. This is in agreement with the blood transcriptomic study in humans [18] in which expression of a cluster of genes relating to the actin cytoskeleton and focal adhesion also increased with ageing (*ACTA2*, *ACTN4*, *CSRP1*, *ILK*, *LPP*, *TAGLIN*, *TLN1*, *VCL* and *WDR1*). In contrast, *ARHGAP15* was more highly expressed in younger cows (this study) and in humans [18]. This gene encodes Rho GTPase activating protein 15 which is involved in T- and B-cell signalling and promotes an increase in actin stress fibres and cell contraction [71].

## Conclusions

The samples collected for this study provided the opportunity to analyse the transcriptomic profile of blood leukocytes in a large number of early lactation cows and we were thus able to capture the complex and temporally dynamic biological pathways which alter as cows age. We have, we believe for the first time, identified genes and pathways associated with increasing lactation number in cows and found that many were comparable to those known to be associated with ageing in humans and model organisms. Immune-related pathways were differentially expressed between primiparous and older cows, including genes involved in innate and adaptive immunity, with many immune defence genes being more highly expressed in MP > 3 animals. In addition, we found changes in mitochondrial and metabolic function, ribosome biogenesis, transcriptional regulation and DNA replication, elongation and repair. Pathways supplying leukocytes with energy changed in cows with different lactation numbers, with increased expression of genes encoding enzymes involved in beta-oxidation of fatty acids in the PP cows whereas genes involved in glycolysis were up-regulated in the older cows. These changes may be particularly relevant to understanding how dairy cows respond to metabolic stress during early lactation, when they are short of energy and there is competition for nutrient supply between the demands of milk production and the need for immune defence. An improved understanding of these processes may help dairy farmers to improve both genetic selection and nutritional management to increase longevity, so benefitting agricultural production and animal welfare.

## Methods

### GPlusE study design and sample collection

The samples used in this study were collected as a part of the Genotype plus Environment (GplusE) FP7-Project (http://www.gpluse.eu). The animals were located on six experimental dairy farms belonging to members of the consortium (Supplementary Table 1). One 3 ml blood sample from each cow was drawn from the tail vein into a Tempus blood collection tubes (Thermo Fischer, UK) in early lactation, at around 14 days after calving and stored at − 20 °C. This sample was taken with the informed consent and ethical approval of the organisations involved and complied with the relevant national and EU legislation under the European Union (Protection of Animals used for Scientific Purposes) Regulations 2012 (S.I. No. 543 of 2012). All cows were subsequently released back into the herd. Details of the management of each herd and their average milk yields over the first 50 days in milk are provided in Krogh et al. [72].

A total of 229 adult Holstein Friesian cows ranging between parities 1 and 7 were recruited. These were subsequently divided into three lactation number groups for analysis: i) primiparous (PP, *n* = 53), ii) multiparous in lactations 2–3 (MP 2–3, *n* = 121), and iii) MP in lactations 4–7 (MP > 3, *n* = 55).

### RNA isolation, library preparation, and sequencing

The individuals performing the sample processing were blinded to the lactation groups. Total RNA was isolated from whole blood leukocytes using the Tempus Spin RNA isolation Kit (Thermo Fischer, Loughborough, UK) following the manufacturer’s instructions. Sample purity and RNA quantity were measured using both a NanoDrop 1000 (Thermo Fischer, UK) and an Agilent BioAnalyzer 2000 using the Agilent RNA 6000 Nano Kit (Agilent, Cheadle, UK; Supplementary Table 6). No samples were excluded based on RNA quality. Library preparation was conducted at the University of Liege (GIGA Research Facility, Liege, Belgium), using 0.75 μg of total RNA with the Illumina TruSeq Stranded Total RNA Ribo-Zero Gold Sample Preparation kit (Illumina, San Diego, USA) and sequenced on the Illumina NextSeq 500 sequencer, producing on average 30 million single end reads of 75 nucleotides length per sample.

### Transcriptomic analyses

Reads with poor quality were trimmed or removed according to base quality using Trimmomatic v.0.36 [73]. The quality of raw and trimmed FASTQ files was assessed with FastQC (http://www.bioinformatics.babraham.ac.uk/projects/fastqc/). The latest version of the *Bos taurus* assembly (ARS-UCD1.2), and its corresponding gene set, was used as reference to map reads using the splice aware aligner HISAT2 [74]. Next, SAM files were converted to BAM files and coordinate sorted with SAMtools [75]. BAM files were processed further with Picard Tools (http://picard.sourceforge.net/) in order to remove PCR duplicates, add read group information, sort by chromosome and create indexes. Reads per gene were counted with StringTie [76].

Differential leukocyte gene expression analysis between the three lactation number groups was conducted with the package DESeq2 [77]. Herd effect was considered and removed using *limma* remove batch effect (limma::removeBatchEffect; Supplementary Fig. 3). Weighted gene co-expression network analysis (WGCNA, R package [78]; was used to construct a co-expression network on the DESeq2 normalized data. WGCNA follows a 6-step process to predict which genes are connected to each other, cluster them into gene networks and test which gene networks are associated with phenotypic status, leading to the selection of hub genes.

Genes with variance < 0.05 were filtered out, and the results (total of 13,769 genes) were used as input to the signed WGCNA network construction. We generated a “signed weighted” adjacency matrix for downstream analyses in which the direction of a pair of genes’ correlation is preserved, and a positive correlation may indicate “activation” whereas a negative correlation may indicate “repression”. The adjacency matrix reported a correlation value between every pair of gene expression measurement across all 229 samples. The next step was to raise the adjacency matrix to a software-determined exponential power, thereby reducing noise by pushing weaker pairwise connection values closer to zero relative to stronger values. The exponential power used is the lowest value needed to ensure the network approximates scale-free topology and was set to seven. The adjacency matrix was then transformed into a “topological overlap” matrix by calculating topological overlap (TOM) scores for each gene. This score accounts for each pair of genes’ connection strength (adjacency value) to each other as well as their connection strengths (adjacency values) to every other gene in the adjacency matrix. WGCNA identifies gene co-expression networks via average linkage hierarchical clustering using a TOM-based dissimilarity measure as a measure of distance. The resultant dendrogram of clustered genes is segregated into individual modules with at least 35 genes using WGCNA’s dynamic tree-cutting algorithm. WGCNA calculates each module’s “eigengene” (first principle component), using all samples’ gene expression values for all genes in that module. A module eigengene is considered a summarized expression profile representative of that module for all samples. Finally, each module eigengene was tested for statistical association to the phenotypic trait of lactation number (PP, MP2–3 and MP > 3).

Cattle genes based on the Ensembl v.100 database were retrieved using the Ensembl BioMart online tool [79] and gene ontology (GO) analysis was performed using genes found in significantly correlated modules to trait. Moreover, a David functional annotation cluster analysis [27] was performed; all 13,769 genes were used as background against all significantly correlated modules.

## Supplementary Information

**Additional file 1: ** Additional tables supporting the main text of the manuscript. Referred to as Supplementary Tables 1–6. **Supplementary Table 1**. Milk yield data (kg/d) for the six dairy herds used in the study, organised by herd and parity. **Supplementary Table 2**. Mapping rate of all blood samples against the *Bos taurus* reference ARS-UCD1.2 and summary statistics. **Supplementary Table 3**. Gene ontology enrichment analysis of genes in significantly correlated modules. **Supplementary Table 4**. David functional cluster analysis of genes in significantly correlated modules. **Supplementary Table 5**A, 5B. (i) Top six most significant clusters; (ii) Number of genes highly expressed in the PP/MP3 cows per cluster. **Supplementary Table 6**. RNA concentration measured with Nanodrop, Bioanalyzer, and RIN values.

**Additional file 2: Supplementary Fig. 1.** Additional figures supporting the main text of the manuscript. Referred to as Supplementary Figs. 1–3. Supplementary Fig. 1. Volcano plot of differentially expressed genes (padj < 0.01, LFC > 1). **Supplementary Fig. 2**. Outliers detected by sample clustering (WGCNA). **Supplementary Fig. 3**. Principal component analysis of herd effect. A) with herd effect, B) herd effect removed.

## Data Availability

The datasets generated and analysed during the current study are available in the NCBI BioProject database under the accession number PRJNA749896.

## Abbreviations

MP: Multiparous
PP: Primiparous
WGCNA: Weighted gene co-expression network analysis
NEFA: Nonesterified fatty acid
BHB: Beta-hydroxybutyrate
DEG: Differentially expressed genes
GO: Gene ontology
PRR: Pattern recognition receptors
PBMC: Peripheral blood mononuclear cell

## References

[1] Pritchard T, Coffey M, Mrode R, Wall E (2013). Understanding the genetics of survival in dairy cows. J Dairy Sci.

[2] De Vries A, Marcondes MI (2020). Review: overview of factors affecting productive lifespan of dairy cows. Anim Int J Anim Biosci.

[3] Dallago GM, Wade KM, Cue RI, McClure JT, Lacroix R, Pellerin D (2021). Keeping dairy cows for longer: a critical literature review on dairy cow longevity in high milk-producing countries. Animals (Basel).

[4] Boulton AC, Rushton J, Wathes DC (2017). An empirical analysis of the cost of rearing dairy heifers from birth to first calving and the time taken to repay these costs. Anim Int J Anim Biosci.

[5] Ray DE, Halbach TJ, Armstrong DV (1992). Season and lactation number effects on milk production and reproduction of dairy cattle in Arizona. J Dairy Sci.

[6] Dennis NA, Stachowicz K, Visser B, Hely FS, Berg DK, Friggens NC, Amer PR, Meier S, Burke CR (2018). Combining genetic and physiological data to identify predictors of lifetime reproductive success and the effect of selection on these predictors on underlying fertility traits. J Dairy Sci.

[7] Grandl F, Furger M, Kreuzer M, Zehetmeier M (2019). Impact of longevity on greenhouse gas emissions and profitability of individual dairy cows analysed with different system boundaries. Anim Int J Anim Biosci.

[8] Esslemont RJ, Kossaibati MA (1997). Culling in 50 dairy herds in England. Vet Rec.

[9] Bell MJ, Wall E, Russell G, Roberts DJ, Simm G (2010). Risk factors for culling in Holstein-Friesian dairy cows. Vet Rec.

[10] De Vries A (2020). Symposium review: why revisit dairy cattle productive lifespan?. J Dairy Sci.

[11] Drackley JK, Overton TR, Douglas GN (2001). Adaptations of glucose and long-chain fatty acid metabolism in liver of dairy cows during the periparturient period. J Dairy Sci.

[12] Wathes DC, Fenwick M, Cheng Z, Bourne N, Llewellyn S, Morris DG, Kenny D, Murphy J, Fitzpatrick R (2007). Influence of negative energy balance on cyclicity and fertility in the high producing dairy cow. Theriogenology.

[13] Wathes DC, Cheng Z, Bourne N, Taylor VJ, Coffey MP, Brotherstone S (2007). Differences between primiparous and multiparous dairy cows in the inter-relationships between metabolic traits, milk yield and body condition score in the periparturient period. Domest Anim Endocrinol.

[14] Taylor VJ, Cheng Z, Pushpakumara PGA, Beever DE, Wathes DC (2004). Relationships between the plasma concentrations of insulin-like growth factor-I in dairy cows and their fertility and milk yield. Vet Rec.

[15] Wathes DC, Cheng Z, Chowdhury W, Fenwick MA, Fitzpatrick R, Morris DG, Patton J, Murphy JJ (2009). Negative energy balance alters global gene expression and immune responses in the uterus of postpartum dairy cows. Physiol Genomics.

[16] Habel J, Sundrum A. Mismatch of glucose allocation between different life functions in the transition period of dairy cows. Animals (Basel). 2020;10. 10.3390/ani10061028. 10.3390/ani10061028 PMC7341265 32545739

[17] Nikolich-Žugich J (2018). The twilight of immunity: emerging concepts in aging of the immune system. Nat Immunol.

[18] Peters MJ, Joehanes R, Pilling LC, Schurmann C, Conneely KN, Powell J (2015). The transcriptional landscape of age in human peripheral blood. Nat Commun.

[19] Kenyon CJ (2010). The genetics of ageing. Nature.

[20] Tarkhov AE, Alla R, Ayyadevara S, Pyatnitskiy M, Menshikov LI, Shmookler Reis RJ, Fedichev PO (2019). A universal transcriptomic signature of age reveals the temporal scaling of Caenorhabditis elegans aging trajectories. Sci Rep.

[21] Moskalev AA, Shaposhnikov MV, Zemskaya NV, Koval LА, Schegoleva EV, Guvatova ZG, Krasnov GS, Solovev IA, Sheptyakov MA, Zhavoronkov A, Kudryavtseva AV (2019). Transcriptome analysis of long-lived Drosophila melanogaster E(z) mutants sheds light on the molecular mechanisms of longevity. Sci Rep.

[22] Shavlakadze T, Morris M, Fang J, Wang SX, Zhu J, Zhou W (2019). Age-related gene expression signature in rats demonstrate early, late, and linear transcriptional changes from multiple tissues. Cell Rep.

[23] Lai RW, Lu R, Danthi PS, Bravo JI, Goumba A, Sampathkumar NK, Benayoun BA (2019). Multi-level remodeling of transcriptional landscapes in aging and longevity. BMB Rep.

[24] Frenk S, Houseley J (2018). Gene expression hallmarks of cellular ageing. Biogerontology.

[25] Kirkwood TBL (2005). Understanding the odd science of aging. Cell.

[26] Barzilai N, Gabriely I (2010). Genetic studies reveal the role of the endocrine and metabolic Systems in Aging. J Clin Endocrinol Metab.

[27] Huang DW, Sherman BT, Lempicki RA (2009). Systematic and integrative analysis of large gene lists using DAVID bioinformatics resources. Nat Protoc.

[28] Chou JP, Ramirez CM, Wu JE, Effros RB (2013). Accelerated aging in HIV/AIDS: novel biomarkers of senescent human CD8+ T cells. PLoS One.

[29] Harries LW, Fellows AD, Pilling LC, Hernandez D, Singleton A, Bandinelli S, Guralnik J, Powell J, Ferrucci L, Melzer D (2012). Advancing age is associated with gene expression changes resembling mTOR inhibition: evidence from two human populations. Mech Ageing Dev.

[30] Passtoors WM, Boer JM, Goeman JJ, van den Akker EB, Deelen J, Zwaan BJ, Scarborough A, van der Breggen R, Vossen RHAM, Houwing-Duistermaat JJ, van Ommen GJB, Westendorp RGJ, van Heemst D, de Craen AJM, White AJ, Gunn DA, Beekman M, Slagboom PE (2012). Transcriptional profiling of human familial longevity indicates a role for ASF1A and IL7R. PLoS One.

[31] Newman AB, Murabito JM (2013). The epidemiology of longevity and exceptional survival. Epidemiol Rev.

[32] Yu C-E, Oshima J, Fu Y-H, Wijsman EM, Hisama F, Alisch R, Matthews S, Nakura J, Miki T, Ouais S, Martin GM, Mulligan J, Schellenberg GD (1996). Positional cloning of the Werner’s gyndrome gene. Science.

[33] Houtkooper RH, Mouchiroud L, Ryu D, Moullan N, Katsyuba E, Knott G, Williams RW, Auwerx J (2013). Mitonuclear protein imbalance as a conserved longevity mechanism. Nature.

[34] de Magalhães JP, Curado J, Church GM (2009). Meta-analysis of age-related gene expression profiles identifies common signatures of aging. Bioinformatics.

[35] Sebastiani P, Montano M, Puca A, Solovieff N, Kojima T, Wang MC (2009). RNA editing genes associated with extreme old age in humans and with lifespan in C elegans. PloS One.

[36] Luo J (2010). WRN protein and Werner syndrome. North Am J Med Sci.

[37] Zhang Q, Guldbrandtsen B, Thomasen JR, Lund MS, Sahana G (2016). Genome-wide association study for longevity with whole-genome sequencing in 3 cattle breeds. J Dairy Sci.

[38] Bell AW, Bauman DE (1997). Adaptations of glucose metabolism during pregnancy and lactation. J Mammary Gland Biol Neoplasia.

[39] Brickell JS, Wathes DC (2011). A descriptive study of the survival of Holstein-Friesian heifers through to third calving on English dairy farms. J Dairy Sci.

[40] Roland L, Drillich M, Iwersen M (2014). Hematology as a diagnostic tool in bovine medicine. J Vet Diagn Investig Off Publ Am Assoc Vet Lab Diagn Inc.

[41] Blankley S, Berry MPR, Graham CM, Bloom CI, Lipman M, O’Garra A (2014). The application of transcriptional blood signatures to enhance our understanding of the host response to infection: the example of tuberculosis. Philos Trans R Soc B Biol Sci.

[42] Franceschi C, Garagnani P, Vitale G, Capri M, Salvioli S (2017). Inflammaging and “Garb-aging.”. Trends Endocrinol Metab.

[43] Akbar AN, Gilroy DW (2020). Aging immunity may exacerbate COVID-19. Science.

[44] Landis G, Shen J, Tower J (2012). Gene expression changes in response to aging compared to heat stress, oxidative stress and ionizing radiation in Drosophila melanogaster. Aging.

[45] Moro-García MA, Alonso-Arias R, López-Larrea C (2012). Molecular mechanisms involved in the aging of the T-cell immune response. Curr Genomics.

[46] Cheng Z, Wylie A, Ferris C, Ingvartsen KL, Wathes DC, GplusE Consortium. Effect of diet and nonesterified fatty acid levels on global transcriptomic profiles in circulating peripheral blood mononuclear cells in early lactation dairy cows. J Dairy Sci. 2021;104(9):10059-75. 10.3168/jds.2021-20136 34147225

[47] Berry MPR, Graham CM, McNab FW, Xu Z, Bloch SAA, Oni T (2010). An interferon-inducible neutrophil-driven blood transcriptional signature in human tuberculosis. Nature.

[48] Park H-E, Park H-T, Jung YH, Yoo HS (2018). Gene expression profiles of immune-regulatory genes in whole blood of cattle with a subclinical infection of Mycobacterium avium subsp paratuberculosis. PLOS ONE.

[49] Campisi J, Kapahi P, Lithgow GJ, Melov S, Newman JC, Verdin E (2019). From discoveries in ageing research to therapeutics for healthy ageing. Nature.

[50] Shigenaga MK, Hagen TM, Ames BN (1994). Oxidative damage and mitochondrial decay in aging. Proc Natl Acad Sci U S A.

[51] Bratic A, Larsson N-G (2013). The role of mitochondria in aging. J Clin Invest.

[52] Morimoto RI, Cuervo AM (2014). Proteostasis and the aging proteome in health and disease. J Gerontol A Biol Sci Med Sci.

[53] van Dorp RT, Martin SW, Shoukri MM, Noordhuizen JP, Dekkers JC (1999). An epidemiologic study of disease in 32 registered Holstein dairy herds in British Columbia. Can J Vet Res Rev Can Rech Veterinaire.

[54] Bettedi L, Foukas LC (2017). Growth factor, energy and nutrient sensing signalling pathways in metabolic ageing. Biogerontology.

[55] De Bont R, van Larebeke N (2004). Endogenous DNA damage in humans: a review of quantitative data. Mutagenesis.

[56] Kenyon J, Gerson SL (2007). The role of DNA damage repair in aging of adult stem cells. Nucleic Acids Res.

[57] Hoeijmakers JHJ (2009). DNA damage, aging, and cancer. N Engl J Med.

[58] Seeker LA, Underwood SL, Wilbourn RV, Dorrens J, Froy H, Holland R, Ilska JJ, Psifidi A, Bagnall A, Whitelaw B, Coffey M, Banos G, Nussey DH (2021). Telomere attrition rates are associated with weather conditions and predict productive lifespan in dairy cattle. Sci Rep.

[59] Aihara K, Kuroda S, Kanayama N, Matsuyama S, Tanizawa K, Horie M (2003). A neuron-specific EGF family protein, NELL2, promotes survival of neurons through mitogen-activated protein kinases. Brain Res Mol Brain Res.

[60] Munemasa Y, Chang C-S, Kwong JMK, Kyung H, Kitaoka Y, Caprioli J, Piri N (2012). The neuronal EGF-related gene Nell2 interacts with Macf1 and supports survival of retinal ganglion cells after optic nerve injury. PLoS One.

[61] Kim DY, Kim HR, Kim KK, Park JW, Lee BJ (2015). NELL2 function in the protection of cells against endoplasmic seticulum stress. Mol Cells.

[62] Johnson SC, Rabinovitch PS, Kaeberlein M (2013). mTOR is a key modulator of ageing and age-related disease. Nature.

[63] Smith HJ, Sharma A, Mair WB (2020). Metabolic communication and healthy aging: where should we focus our energy?. Dev Cell.

[64] Steri R, Moioli B, Catillo G, Galli A, Buttazzoni L (2019). Genome-wide association study for longevity in the Holstein cattle population. Anim Int J Anim Biosci.

[65] Zou Y, Gong N, Cui Y, Wang X, Cui A, Chen Q, Jiao T, Dong X, Yang H, Zhang S, Fang F, Chang Y (2015). Forkhead box P1 (FOXP1) transcription factor regulates hepatic glucose homeostasis. J Biol Chem.

[66] Mu Z, Wang L, Deng W, Wang J, Wu G (2017). Structural insight into the Ragulator complex which anchors mTORC1 to the lysosomal membrane. Cell Discov.

[67] Mancarella C, Scotlandi K (2019). IGF2BP3 from physiology to cancer: novel discoveries, unsolved issues, and future perspectives. Front Cell Dev Biol.

[68] Walls J, Sinclair L, Finlay D (2016). Nutrient sensing, signal transduction and immune responses. Semin Immunol.

[69] Crookenden MA, Moyes KM, Kuhn-Sherlock B, Lehnert K, Walker CG, Loor JJ, Mitchell MD, Murray A, Dukkipati VSR, Vailati-Riboni M, Heiser A, Roche JR (2019). Transcriptomic analysis of circulating neutrophils in metabolically stressed peripartal grazing dairy cows. J Dairy Sci.

[70] Minuti A, Jahan N, Lopreiato V, Piccioli-Cappelli F, Bomba L, Capomaccio S, Loor JJ, Ajmone-Marsan P, Trevisi E (2020). Evaluation of circulating leukocyte transcriptome and its relationship with immune function and blood markers in dairy cows during the transition period. Funct Integr Genomics.

[71] Seoh ML, Ng CH, Yong J, Lim L, Leung T (2003). ArhGAP15, a novel human RacGAP protein with GTPase binding property. FEBS Lett.

[72] Krogh MA, Hostens M, Salavati M, Grelet C, Sorensen MT, Wathes DC, Ferris CP, Marchitelli C, Signorelli F, Napolitano F, Becker F, Larsen T, Matthews E, Carter F, Vanlierde A, Opsomer G, Gengler N, Dehareng F, Crowe MA, Ingvartsen KL, Foldager L (2020). Between- and within-herd variation in blood and milk biomarkers in Holstein cows in early lactation. Animal.

[73] Bolger AM, Lohse M, Usadel B (2014). Trimmomatic: a flexible trimmer for Illumina sequence data. Bioinforma Oxf Engl.

[74] Kim D, Paggi JM, Park C, Bennett C, Salzberg SL (2019). Graph-based genome alignment and genotyping with HISAT2 and HISAT-genotype. Nat Biotechnol.

[75] Li H, Handsaker B, Wysoker A, Fennell T, Ruan J, Homer N, Marth G, Abecasis G, Durbin R, 1000 Genome Project Data Processing Subgroup (2009). The sequence alignment/map format and SAMtools. Bioinforma Oxf Engl.

[76] Pertea M, Pertea GM, Antonescu CM, Chang T-C, Mendell JT, Salzberg SL (2015). StringTie enables improved reconstruction of a transcriptome from RNA-seq reads. Nat Biotechnol.

[77] Love MI, Huber W, Anders S (2014). Moderated estimation of fold change and dispersion for RNA-seq data with DESeq2. Genome Biol.

[78] Langfelder P, Horvath S (2008). WGCNA: an R package for weighted correlation network analysis. BMC Bioinformatics.

[79] Smedley D, Haider S, Ballester B, Holland R, London D, Thorisson G, Kasprzyk A (2009). BioMart--biological queries made easy. BMC Genomics.

