# Supplementary material for: Comparison of the transcriptome in circulating leukocytes in early lactation between primiparous and multiparous cows provides evidence for age-related changes
Source: BMC Genomics. 2021 Sep 25;22:693. doi: 10.1186/s12864-021-07977-5 (PMC8466696; doi:10.1186/s12864-021-07977-5)

Supplementary Figure 1. Volcano plot of differentially expressed genes (padj <0.01, LFC>1).

PP vs MP>3

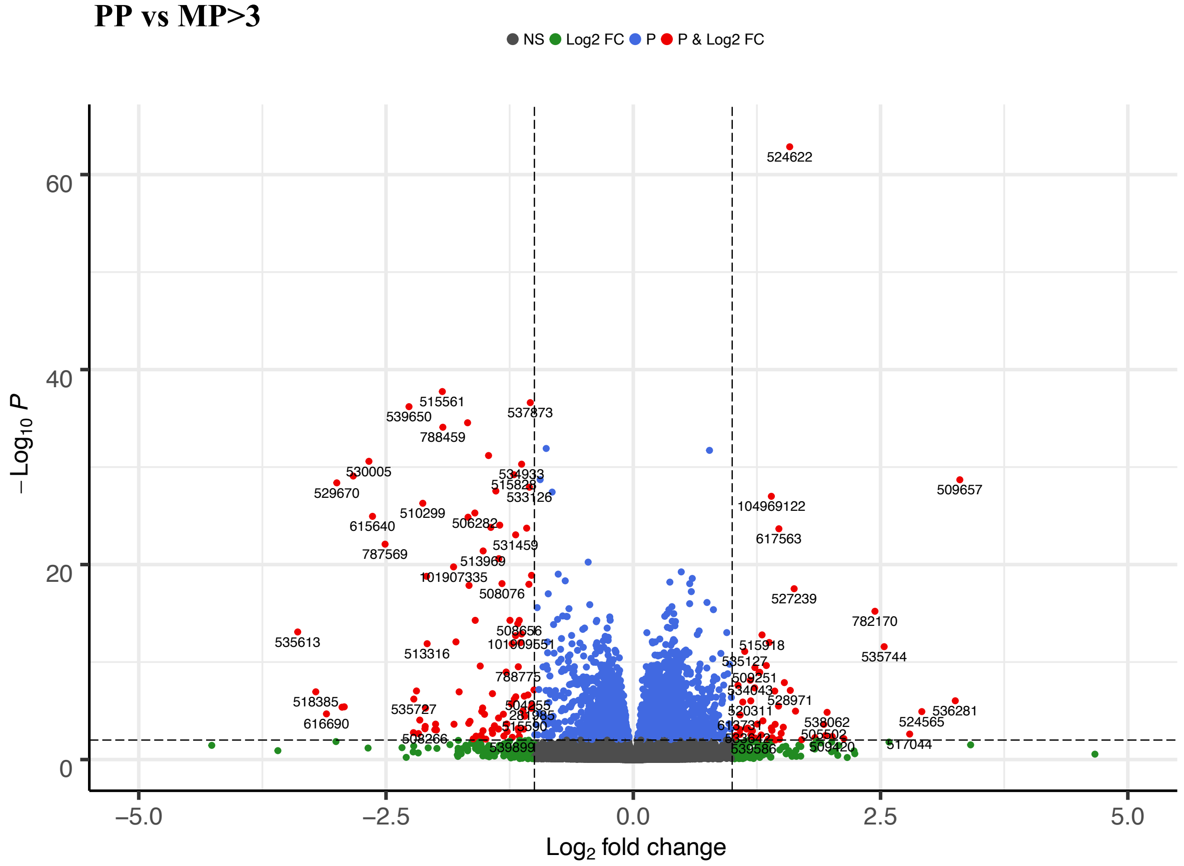

Supplementary Figure 2. Outlier detected by sample clustering (WGCNA).

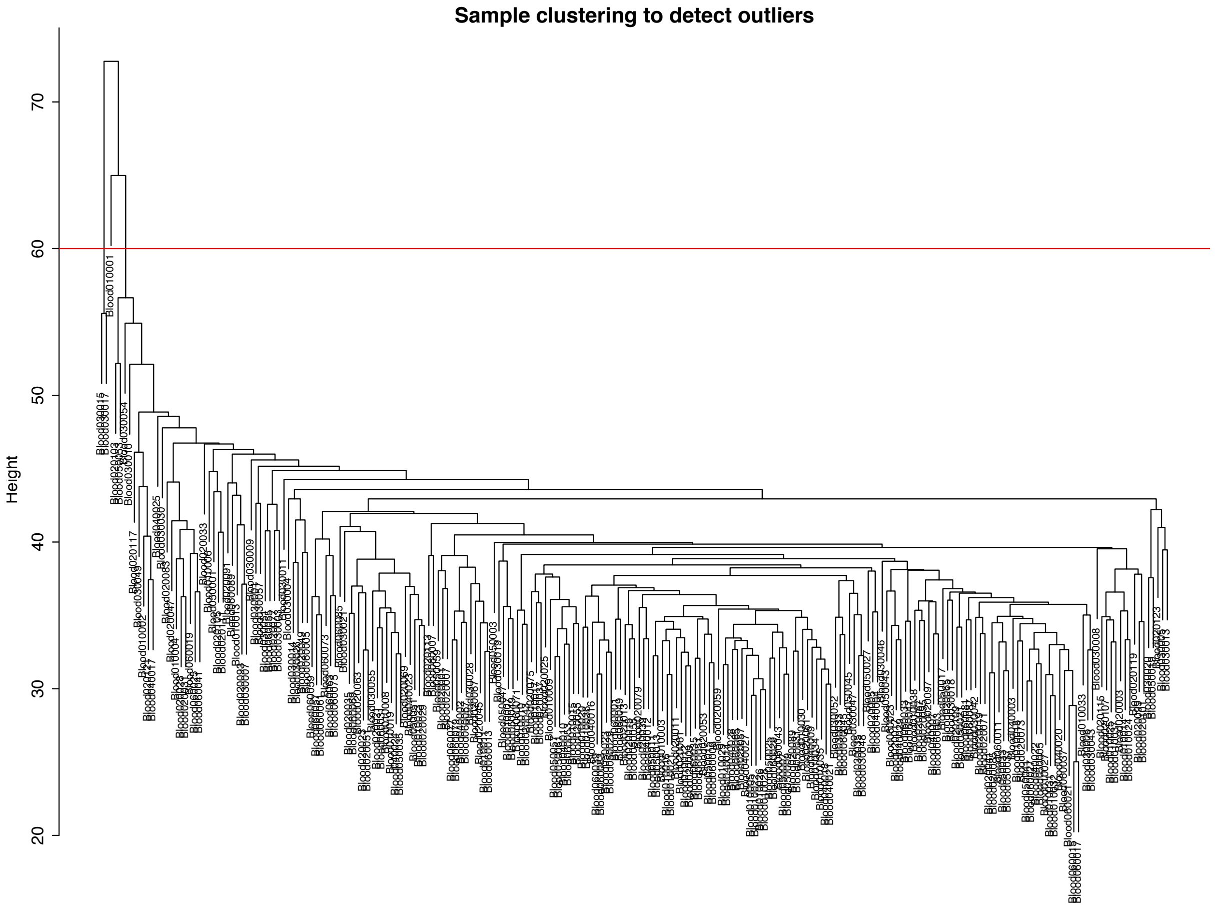

Supplementary Figure 3. Principal component analysis of herd effect. A) with herd effect, B) herd effect removed

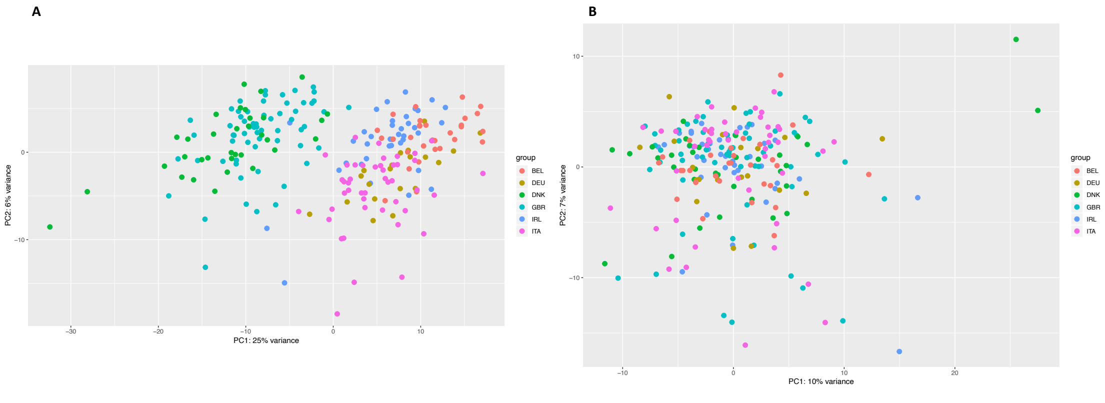

Supplement: Supplementary file 2 — Additional file 2: Supplementary Fig. 1. Additional figures supporting the main text of the manuscript. Referred to as Supplementary Figs. 1–3. Supplementary Fig. 1. Volcano plot of differentially expressed genes (padj < 0.01, LFC > 1). Supplementary Fig. 2. Outliers detected by sample clustering (WGCNA). Supplementary Fig. 3. Principal component analysis of herd effect. A) with herd effect, B) herd effect removed. [file 12864_2021_7977_MOESM2_ESM.pdf]
